# Optical Constants of Chemical Vapor Deposited Graphene for Photonic Applications

**DOI:** 10.3390/nano11051230

**Published:** 2021-05-07

**Authors:** Marwa A. El-Sayed, Georgy A. Ermolaev, Kirill V. Voronin, Roman I. Romanov, Gleb I. Tselikov, Dmitry I. Yakubovsky, Natalia V. Doroshina, Anton B. Nemtsov, Valentin R. Solovey, Artem A. Voronov, Sergey M. Novikov, Andrey A. Vyshnevyy, Andrey M. Markeev, Aleksey V. Arsenin, Valentyn S. Volkov

**Affiliations:** 1 Center for Photonics and 2D Materials, Moscow Institute of Physics and Technology, 9 Institutsky Lane, 141700 Dolgoprudny, Russia; mira@phystech.edu (M.A.E.-S.); georgiy.ermolayev@phystech.edu (G.A.E.); voronin.kv@phystech.edu (K.V.V.); tselikov.gi@mipt.ru (G.I.T.); dmitrii.yakubovskii@phystech.edu (D.I.Y.); doroshina.nv@phystech.edu (N.V.D.); nemtsov@phystech.edu (A.B.N.); solovey.vr@phystech.edu (V.R.S.); voronov.aa@mipt.ru (A.A.V.); novikov.s@mipt.ru (S.M.N.); andrey.vyshnevyy@phystech.edu (A.A.V.); markeev.am@mipt.ru (A.M.M.); arsenin.av@mipt.ru (A.V.A.); 2 Department of Physics, Faculty of Science, Menoufia University, Shebin El-Koom 32511, Egypt; 3 Skolkovo Institute of Science and Technology, 3 Nobel, 143026 Moscow, Russia; 4 Moscow Engineering Physics Institute, National Research Nuclear University MEPhI, 31 Kashirskoe Sh., 115409 Moscow, Russia; limpo2003@mail.ru; 5 GrapheneTek, Skolkovo Innovation Center, 143026 Moscow, Russia

**Keywords:** graphene, optical constants, dielectric properties, refractive index, nanophotonics, spectroscopic ellipsometry

## Abstract

Graphene is a promising building block material for developing novel photonic and optoelectronic devices. Here, we report a comprehensive experimental study of chemical-vapor deposited (CVD) monolayer graphene’s optical properties on three different substrates for ultraviolet, visible, and near-infrared spectral ranges (from 240 to 1000 nm). Importantly, our ellipsometric measurements are free from the assumptions of additional nanometer-thick layers of water or other media. This issue is critical for practical applications since otherwise, these additional layers must be included in the design models of various graphene photonic, plasmonic, and optoelectronic devices. We observe a slight difference (not exceeding 5%) in the optical constants of graphene on different substrates. Further, the optical constants reported here are very close to those of graphite, which hints on their applicability to multilayer graphene structures. This work provides reliable data on monolayer graphene’s optical properties, which should be useful for modeling and designing photonic devices with graphene.

## 1. Introduction

Graphene is one of the most attractive materials for the development of promising new photonic, plasmonic, and optoelectronic devices [1,2,3,4,5,6,7]. In particular, graphene is a critical component of nanoscale broadband optical modulators [8,9,10], chip-integrated ultrafast photodetectors [11], highly sensitive and selective sensors [12,13], transparent, flexible solar cells [14,15], among others. The design, simulation, and optimization of such functional components and devices all require knowledge of graphene’s optical constants. A precise method for determining refractive indices and extinction coefficients is spectroscopic ellipsometry, which allows for extracting the dielectric function in a broad wavelength range directly from the raw data [16,17]. The previous works report ellipsometric studies of the optical constants of exfoliated graphene [18,19,20,21,22], epitaxial graphene [23,24,25], and CVD graphene [26,27,28,29,30,31], also transferred onto various substrates such as optical glass [26], silicon oxide [18,20,31], or fused silica [18,19,29,30]. However, the measured dielectric functions show more than 20% differences, caused not only by the graphene production technique, the effect of the substrate, or the quality of graphene but also by the use of different ellipsometric models and respective initial assumptions. For instance, in the analysis of optical properties, one can take into account the adsorption of water [18,28,30,31,32] and polymer residues [18,19] on graphene used for the transfer of both CVD and exfoliated graphene. Therefore, despite extensive research efforts devoted to studying monolayer graphene’s optical response, optical constants’ choice remains challenging due to the lack of consensus in the field.

It is well-known that graphene is easily contaminated by adsorbing water and other organic and inorganic compounds [33,34], but, in most cases, it is meticulously cleaned from contamination and protected from the external environment by encapsulation layers [35]. However, even with thorough cleaning and annealing of graphene from contamination, trace amounts of water or organic molecules may remain. One way to overcome this issue is to include residual contaminants as additional layers in an ellipsometric model to obtain the optical properties of pure graphene. Yet, this approach has its drawbacks. Firstly, one should know the exact amount of contamination, which is very difficult to determine. Furthermore, even if the amount of residues is determined, it is not guaranteed that their influence on graphene sample’s properties can be properly taken into account by adding auxiliary planar layers. Secondly, to assess the expected performance of graphene-based photonic and optoelectronic devices, one should not forget to include these contamination layers in the device model. Additionally, to evaluate the maximum performance of devices, it is essential to investigate the dielectric response of cleaned graphene on typical substrates.

**Figure 1:**
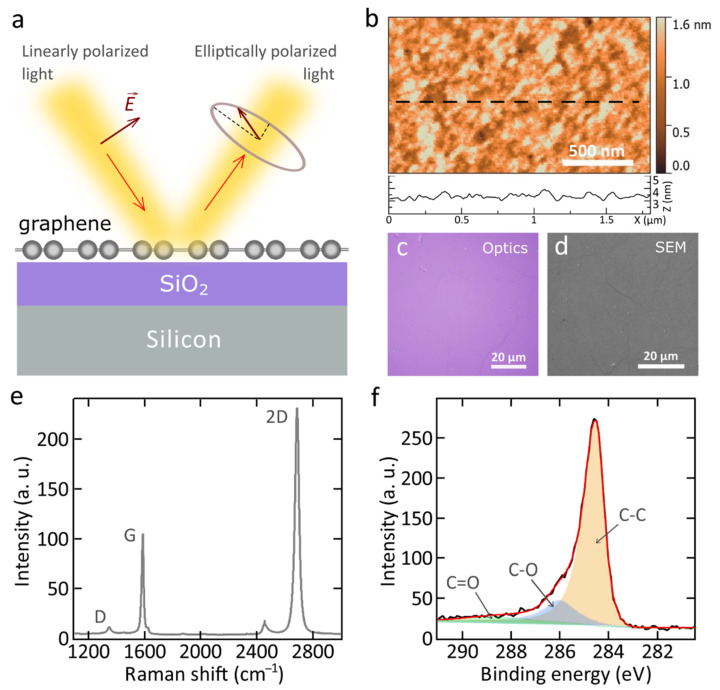
(**a**) The scheme of the ellipsometry measurements. (**b**) AFM topography mapping of single-layer graphene on SiO_2_/Si substrate with line profile across the surface. The scan area was 1.8 × 1.1 μm^2^. (**c**) Optical image of the graphene on top of SiO_2_/Si substrate. (**d**) SEM image of the graphene reveals a high crystallinity of the samples with the crystallite size larger than 10  μm. (**e**) Raman spectrum of graphene transferred on SiO_2_/Si substrate, at excitation wavelength λ = 532 nm. (**f**) Results of XPS fitting analysis of the C1s core level signal obtained for the monolayer graphene on SiO_2_/Si.

Here, we present the optical properties of commercially available monolayer CVD graphene on glass, quartz, and SiO_2_/Si substrates. The optical properties were investigated by highly accurate variable-angle spectroscopic ellipsometry (Figure 1a). Before characterization, graphene samples were washed and annealed in a vacuum chamber to remove polymer residuals and water. Ellipsometry data were analyzed and fitted without any additional assumptions such as the presence of water or any other medium on or under the graphene. To ensure the accuracy of our ellipsometric analysis, we utilized optical transmission spectroscopy measurements and X-ray photoelectron spectroscopy (XPS) to analyze the elemental composition and contribution of residues from the transfer process on the graphene samples. All graphene samples were synthesized by the same method and the same manufacturer, so any discrepancies in optical response should not be related to graphene quality differences. For additional analysis, we performed Raman spectroscopy, atomic-force, and scanning electron microscopy to evaluate the quality, thickness, and uniformity of CVD graphene on different substrates.

## 2. Results and Discussion

### 2.1. Sample Preparation and Characterization

**Figure A1:**
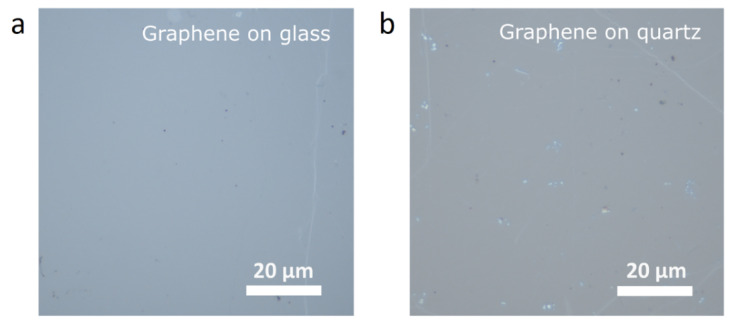
Optical images of the graphene on top of the glass (**a**) and quartz (**b**) substrates.

**Figure A2:**
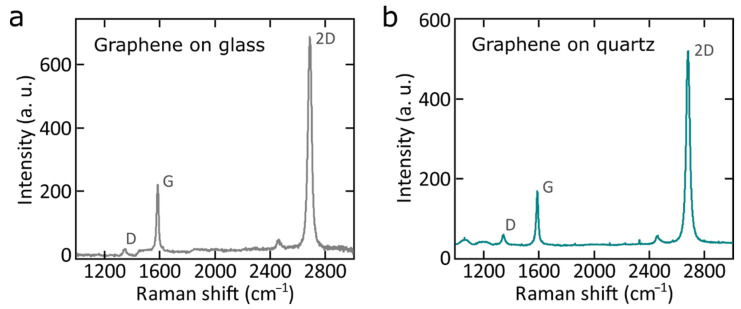
Raman spectra of graphene transferred on glass (**a**) and quartz (**b**) substrates, obtained with an excitation wavelength of λ = 532 nm.

All monolayer graphene films were prepared (see Methods) through chemical vapor deposition. Monolayers of graphene grown initially on copper foil were wet-transferred to three various substrates: SiO_2_ (285 nm)/Si, fused quartz, and optical glass. Before measurements, graphene samples were washed and annealed in a vacuum chamber to remove polymer residuals and water. The high quality of monolayer graphene films was confirmed by atomic force microscopy (AFM), optical microscopy, and scanning electron microscopy (SEM). The AFM scan image of the graphene on SiO_2_/Si in Figure 1b confirms a small surface roughness typical for monolayer graphene surfaces. Large-scale optical microscopy images in Figure 1c and Figure A1a,b, and SEM image of the graphene surface in Figure 1d demonstrate that graphene uniformly covers more than 97% of the substrate surface without voids showing good crystallinity of the graphene sample with the crystallite size >10 μm. To characterize the thickness and quality of monolayer graphene on different substrates, we performed Raman spectroscopy measurements (Figure 1e and Figure A2a,b) [36,37]. The ratio of 2D (2689 cm^−1^) and G (1587 cm^−1^) bands (>2) indicates the single layer of graphene, and the low intensity of the D band (1347 cm^−1^), shows its high quality (Figure 1e). Additionally, the 2D band exhibits a sharp Lorentzian peak, specific to a single layer of graphene [38].

**Figure A3:**
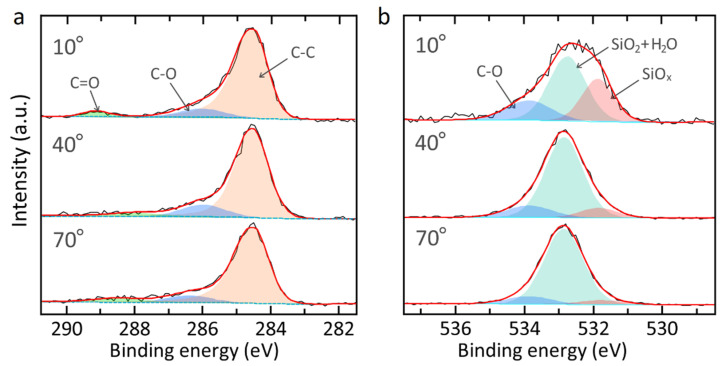
Results of the angle-resolved XPS measurements of the graphene transferred onto SiO_2_/Si substrate. Decomposition of C1s (**a**) and O1s (**b**) core level signals into their constituents. Take-off angle (measured from the sample surface) increases from 10° to 70°; correspondingly, the explored depth of the sample is increased.

Next, to analyze the presence of residues (from the transfer process) and their contribution to the accuracy of graphene dielectric response characterization, we performed X-ray photoelectron spectroscopy. Figure 1f shows the analysis of the C1s core level constituents for the graphene on SiO_2_/Si sample, in which the percentage contribution of sp2-graphene, C–O and C=O bonds to C1s are 80.72%, 14.89% and 4.39%, respectively. To investigate the surface composition on top and under the graphene, we made the angle-resolved XPS measurements for the C1s and O1s core level signals in the range of take-off angles from 10° to 70° (measured from the sample surface) that can change the explored depth (see Figure A3) [28]. Both XPS analyses of the C1s and O1s core level signals in Figure A3 demonstrates a small contribution of C–O and C=O bonds, suggesting the negligible residua of polymer on the graphene surface. As the angle decreases, the relative intensity of the C–O, C=O lines in the decomposition of the C1s spectra and O–C lines in the O1s spectra increases. Consequently, these lines are associated with surface states corresponding to adsorbed molecules and polymethylmethacrylate (PMMA) residues. Oxygen anions in SiO_2_ and H_2_O share the same binding energy region, and their contributions are indistinguishable. However, from the fact that the relative intensity of the SiO_2_ + H_2_O lines in the decomposition of the O1s spectra decreases monotonically with decreasing angle, it can be concluded that H_2_O does not make a significant contribution to the total intensity of these lines. Indeed, as the angle decreases, photoelectrons are detected from a smaller sample depth. Therefore, the contribution from SiO_2_ should decrease. If the water concentration in the sample is significant, it partially compensates for the reduced contribution from SiO_2_. As a result, a nonmonotonic change in the intensity of the SiO_2_ + H_2_O line is observed [28]. Noteworthy, the evolution of constituents of the XPS spectra (Figure A3) with the increasing explored depth did not reveal the presence of contamination interlayers (water, PMMA) [28]. Thus they are not considered in the determination of graphene optical constants.

### 2.2. Dielectric Response Analysis

**Figure 2:**
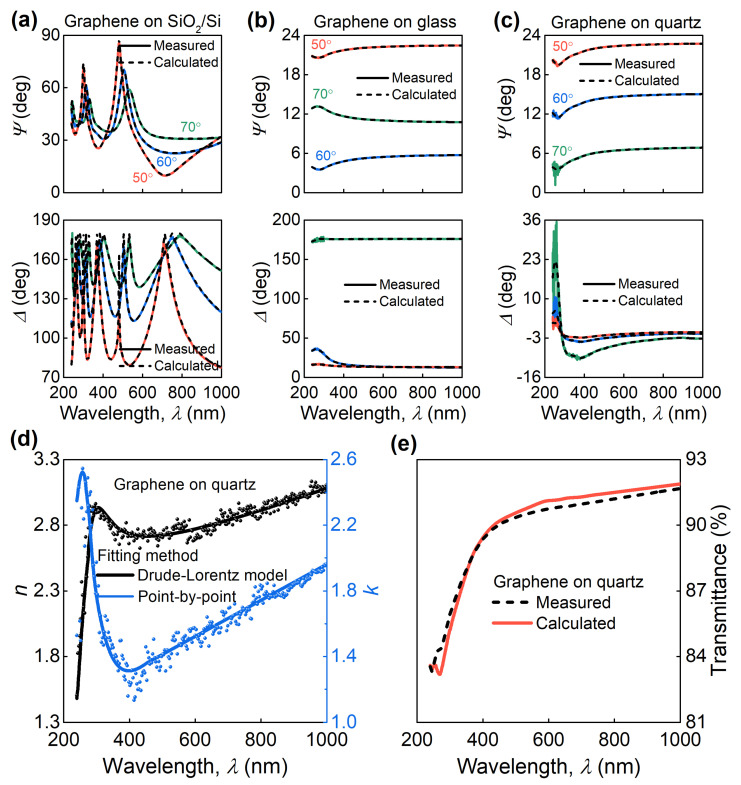
Plots of the measured (experiment) and calculated (model) ellipsometric spectra (Ψ and Δ) of monolayer graphene on different substrates: (**a**) SiO_2_/Si, (**b**) glass, and (**c**) quartz. (**d**) Refractive index *n* and extinction coefficient *k* of monolayer graphene on quartz acquired from SE analysis using different models. (**e**) Measured (red line) and calculated (black dashed line) transmittance spectra match perfectly within spectrophotometer accuracy (1%).

We performed spectroscopic ellipsometry (SE) measurements at multiple incident angles to explore the dielectric response of graphene samples, Figure 2a–c. SE measures the change in light polarization upon reflection from a sample in terms of Ψ and Δ defined through equation tan(Ψ)exp(iΔ) = *r*_p_/*r*_s_, where *r*_p_ and *r*_s_ are sample’s reflection amplitude for p- and s-polarized light. First, we analyzed these Ψ and Δ data by point-by-point inversion to get the initial results presented in Figure 2d. In this approach, optical constants of graphene are varied independently for each wavelength until the best match with the experiment is achieved, allowing to get initial values for the dielectric response of graphene. Then, the dielectric function is fitted by the Drude–Lorentz oscillator model (see Methods and Table 1), which takes into account the optical response of quasi-free electrons (Drude oscillator) and graphene van Hove singularity at π-to-π* interband transition (Lorentz oscillator).Such a fitting approach yields smooth and Kramers–Kronig-consistent dielectric functions compared to noisy point-by-point results (Figure 2d). We calculated the transmittance spectrum based on retrieved optical constants, which is in good agreement with the experimental one as illustrated in Figure 2e, thereby validating the acquired dielectric function.

**Figure 3:**
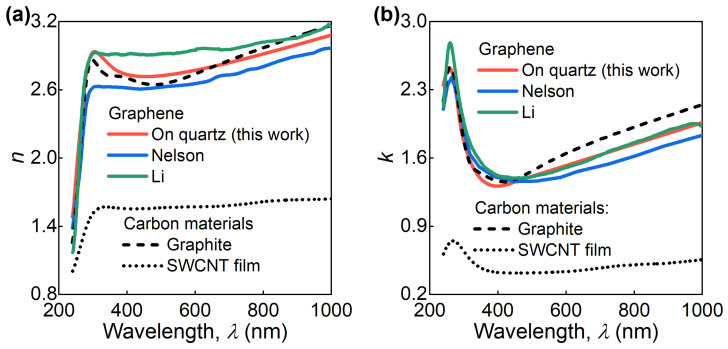
Comparison of the measured (red lines) refractive index *n* (**a**) and extinction coefficient *k* (**b**) and some previous results for CVD graphene on glass (blue lines) and quartz (green lines) substrates obtained without any assumptions in the ellipsometric model and carbon materials such as graphite (dashed lines) and SWCNT film (dotted lines). The data of Nelson, Li, Graphite, and SWCNT film are adopted from refs. [26,29,39,40], respectively.

Interestingly, graphene’s optical response is similar in shape to other carbon materials such as graphite [39] and single-walled carbon nanotube (SWCNT) films [40], as illustrated in Figure 3. More importantly, the optical constants of graphene are almost identical to graphite. Consequently, in the considered wavelength range (240–1000 nm), graphene’s optical response is also similar to any number of graphene layers. As a result, the obtained optical constants of graphene could also be used for bilayer, trilayer, and further graphene layers up to graphite. This observation is in total agreement with the results of previous studies [41].

**Figure 4:**
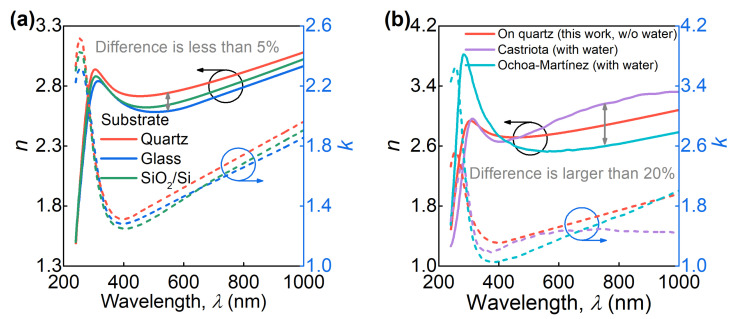
(**a**) The measured refractive index *n* and extinction coefficient *k* of CVD graphene on three different substrates. For the tabular data, see Appendix A Table A1. (**b**) Comparison of the measured optical constants (red lines) and some previous results for CVD graphene obtained under the assumption of the presence of nanometer-thick layers of water in the ellipsometric model (cyan and magenta lines). Solid, *n*, and dashed, *k*, lines. The data of Ochoa-Martinez and Castriota are adopted from refs. [28,31], respectively.

We also investigated graphene transferred on different substrates (quartz, glass, and SiO_2_/Si) to explore substrate effect, which could explain discrepancies observed in the literature for graphene optical constants. We proceeded with the same procedure as for quartz: first, with the point-by-point approach and the Drude–Lorentz model afterward. The resulting parameters for the Drude–Lorentz model and corresponding optical constants for graphene on quartz, glass, and SiO_2_/Si substrates are collected in Table 1 and Figure 4a. Clearly, the dielectric function is reproduced with 95% accuracy. Therefore, the substrate effect is negligible for graphene optical constants.

In contrast, the inclusion of a nanometer-thick water layer in the optical model to consider graphene contamination noticeably changes the result as seen from Figure 4b (there is a large discrepancy by more than 20%). Although this approach allows the inclusion of graphene nonidealities, current devices utilize encapsulated graphene where contamination is minimal. Thus, graphene should be analyzed immediately after the cleaning procedure, as in our work, to enable predictive capabilities for photonic applications.

### 2.3. Applications

**Figure 5:**
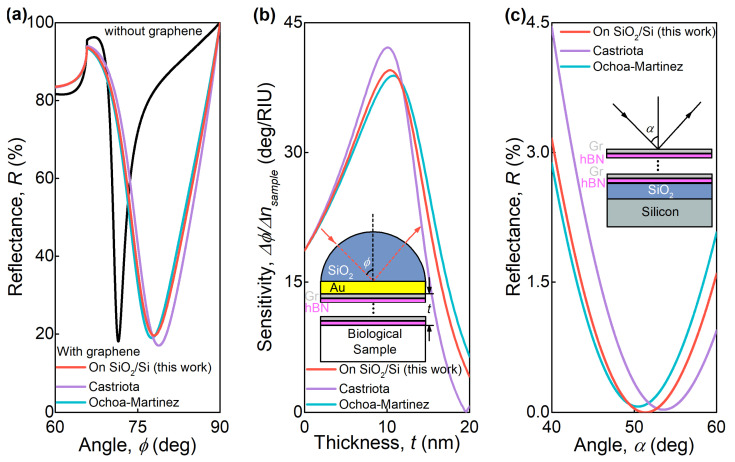
The operation of the SPR sensor based on SiO_2_/Au (Au-thickness equals to 40 nm) chip with the metamaterial, consisting of alternating layers graphene and h-BN (**a**,**b**). (**a**) The dependence of the reflection coefficient as a function of the incident angle at the 800 nm wavelength and 10-nm-thick metamaterial. The black curve corresponds to the absence of the metamaterial layer; red, blue, and green curves calculated using different optical properties of graphene, that were reported. (**b**) The dependence of the sensitivity of this SPR sensor on the thickness of the metamaterial. (**c**) The TM-mode reflectance from the 20-nm-thick metamaterial, consisting of alternating layers graphene and h-BN, placed on the SiO_2_/Si substrate (SiO_2_ thickness equals to 280 nm), as a function of the incident angle at 530 nm free-space wavelength. Red, purple and blue-green curves were calculated using optical properties of graphene from different sources. The optical constants of graphene of Ochoa-Martinez and Castriota are adopted for calculations from refs. [28,31], respectively.

To illustrate the importance of refining the optical constants of graphene, we consider two examples of the devices, including graphene-based heterostructures. One graphene layer could not significantly affect some systems’ optical response because it is too thin, but when several layers of graphene are included in a metamaterial [42,43], the effect of the dielectric function’s inaccuracy becomes apparent. Firstly, we evaluate the optical response of a metamaterial consisting of alternating layers of graphene and h-BN [44], placed on the surface of a standard surface plasmon resonance (SPR) chip, a SiO_2_ prism with a 40-nm-thick gold film [45]. Such metamaterial enhances the SPR chip’s performance due to the rise of optical sensitivity (Figure 5b) and graphene’s ability to effectively adsorb the studied molecules [12,13]. Figure 5a shows the calculated reflectance of the chip with the optimal (see Figure 5b) 10-nm-thick layer of the metamaterial at a free-space wavelength of 800 nm. Evidently, both the resonance depth and position are strongly dependent on the optical properties of graphene. The expected sensitivity of the considered sensor, shown in Figure 5b, varies by more than 10% when different sources of graphene’s optical properties are used in calculations.

In addition, we consider the same metamaterial (Gr/h-BN) with a 20 nm thickness on top of the standard SiO_2_/Si substrate to investigate its zero reflection conditions giving rise to phase singularity used in meta-optics design [46]. We evaluate the incident-angle-dependent reflectance of TM-wave at a wavelength of 530 nm using graphene’s optical properties reported by different sources. For optical constants obtained in our work, this structure exhibits zero reflection at an incident angle of approximately 51 degrees, while in the case of the optical constants obtained in [31], the zero reflection angle shifts to 53 degrees. In contrast, for the optical constants obtained in [28], zero reflection is not achieved.

## 3. Materials and Methods

### 3.1. Materials

Full area coverage graphene samples were purchased from Graphene Laboratories Inc., Ronkonkoma, NY, USA (https://graphene-supermarket.com accessed on 27 April 2021). Graphene was grown via chemical vapor deposition on a copper foil and wet-transferred onto silicon wafer covered by a 285-nm-thick layer of SiO_2_, glass (Corning EAGLE XG, New York, NY, USA), and fused quartz (Volume Precision Glass, Inc. G.E. 124, Santa Rosa, CA, USA) substrates. Monolayer graphene covered about 95% of the substrate area. Before measurements, graphene substrates were washed in acetone and isopropyl alcohol baths and annealed at 200 °C in a vacuum chamber 10^−6^ Torr for more than 1 h to remove residual PMMA and water after the transfer process.

### 3.2. Raman Characterization

The experimental setup used for Raman measurements was a confocal scanning Raman microscope Horiba LabRAM HR Evolution (HORIBA Ltd., Kyoto, Japan). All measurements were carried out using linearly polarized excitation at wavelength 532 nm, 1800 lines/mm diffraction grating, and × 100 objective (N.A. = 0.90). Meanwhile, we used unpolarized detection to have a significant signal-to-noise ratio. The spot size was ~0.4 µm. The Raman spectra were recorded with 0.9 mW incident powers and an integration time of 10 s at each point. The statistics were collected at least 10 points for each sample.

### 3.3. XPS Characterization

For the detailed study of the transferred CVD graphene optical response, we carried out the angle-dependent measurements of the O1s and C1s core level XPS spectra to determine contaminants’ presence on top of graphene. Three take-off angles, 10°, 40° and 70° (measured from the surface of the sample), were used to change the explored depth in XPS measurements. In order to investigate the contributions of different bonds, the core-level spectra of the O1s and C1s were decomposed using the XPS peak fitting software package.

### 3.4. Atomic Force Microscopy, Optical Visualization, Scanning Electron Microscopy

The roughness and homogeneity of the graphene were measured by an atomic force microscope (NT-MDT Ntegra, Moscow, Russia). The surface images (2400 × 2400 pixels) of the graphene samples were captured by an optical microscope (Nikon LV150, Tokyo, Japan) with a digital camera DS-Fi3. To investigate graphene’s surface, we additionally used the scanning electron microscope using the acceleration voltage of 3 kV (JEOL JSM-7001F, Tokyo, Japan).

### 3.5. Ellipsometry Characterization

Spectroscopic ellipsometry was performed at room temperature at multiple incident angles (50°, 60°, 70°), over a broad spectral range from 240 to 1000 nm (1.24–5.17 eV). The measurements were done using a variable-angle spectroscopic ellipsometer (VASE, J.A. Woollam Co., Lincoln, NE, USA). To avoid any crushes or large tears of graphene, we measured samples in different areas, excluding from consideration the areas close to the edges where graphene’s substrate coverage is minimal. For ellipsometry analysis we implemented the Drude–Lorentz model [47]:

$$\begin{array}{l} \varepsilon =\varepsilon_{\mathrm{Drude}}+\varepsilon_{\mathrm{Lorentz}}=\\ =1-\frac{\hbar^{2}}{\varepsilon_{0}\rho (\tau E^{2}+i\hbar E)}+\frac{A_{L}B_{L}E_{L}}{E_{L}^{2}-E^{2}-iB_{L}E}, \end{array}$$

where *ε*_0_ is the vacuum dielectric constant, *ħ* is Planck’s constant, *ρ* is the resistivity, *τ* is the mean scattering time, and *A*_L_, *B*_L_, and *E*_L_ are the amplitude, the linewidth, and the resonance energy of the Lorentz oscillator.

## 4. Conclusions

To summarize, we have presented a UV-visible-near-IR (240–1000 nm) spectroscopic ellipsometry of graphene grown by chemical vapor deposition and transferred onto glass, quartz, and SiO_2_/Si substrates. To obtain an accurate dielectric function suitable for engineering purposes, we thoroughly cleaned and annealed our samples prior to measurements and carried out XPS analysis confirming an ultra-low level of contamination on the graphene’s surface. As a result, we achieved good convergence using a simple ellipsometric model comprising layers of substrate and graphene layer, without auxiliary layers, which are typically introduced to account for residual water and PMMA. The accuracy of the measured optical response was further confirmed by transmittance measurements, which demonstrate remarkable agreement with calculations based on the measured optical constants. Notably, our optical constants show less than 5% variation with changes of the substrate and are very similar to those of graphite, which implies that the measured optical constants are applicable to multilayer graphene structures as well. From a broader perspective, our work provides accurate and universal graphene optical response for the design of photonic devices.

## Figures and Tables

**Table 1:** Drude–Lorentz oscillators parameters for graphene on SiO_2_/Si, quartz, and glass.

Substrate	*A*_L_	*B*_L_ (eV)	*E*_L_ (eV)	*ρ* (10^−4^ Ω∙m)	*τ* (fs)
SiO_2_/Si	8.0531	1.5891	4.5715	5.3668	0.60878
Quartz	8.4404	1.6275	4.6179	4.8038	0.65817
Glass	7.8742	1.5650	4.5201	5.2734	0.61750

## Data Availability

The data presented in this study are available on request from the corresponding author.

## Appendix A

**Table A1:** Tabulated optical constants for graphene on different substrates.

λ (nm)	Substrate					
	SiO_2_/Si		Quartz		Glass	
	*n*	*k*	*n*	*k*	*n*	*k*
240	1.5018	2.3036	1.4913	2.3303	1.5033	2.2261
260	2.1096	2.4266	2.1676	2.5087	2.0678	2.3145
280	2.6262	2.1543	2.7044	2.1765	2.5344	2.1402
300	2.866	1.7567	2.9296	1.7667	2.7947	1.8313
320	2.8629	1.496	2.9093	1.5577	2.8391	1.5565
340	2.8004	1.3584	2.8452	1.4296	2.7962	1.389
360	2.7408	1.2902	2.7901	1.3557	2.7397	1.3133
380	2.6956	1.2595	2.7539	1.3209	2.6909	1.2874
400	2.6637	1.2498	2.7338	1.3126	2.6531	1.2834
420	2.6426	1.2529	2.7222	1.3219	2.6255	1.2907
440	2.6298	1.2639	2.7167	1.343	2.6063	1.3044
460	2.6234	1.28	2.7158	1.3683	2.5938	1.3216
480	2.6221	1.2997	2.7182	1.3905	2.5864	1.3408
500	2.6247	1.3216	2.7232	1.4125	2.5838	1.3609
520	2.6305	1.3451	2.7301	1.4345	2.5849	1.3815
540	2.6389	1.3697	2.7386	1.4565	2.5888	1.4022
560	2.6493	1.395	2.7483	1.4785	2.5952	1.4228
580	2.6614	1.4207	2.7591	1.5006	2.6044	1.4433
600	2.6749	1.4465	2.7708	1.5226	2.6167	1.4635
620	2.6894	1.4725	2.7832	1.5446	2.6316	1.4836
640	2.7049	1.4984	2.7962	1.5666	2.6485	1.5035
660	2.721	1.5243	2.8098	1.5886	2.6663	1.5233
680	2.7376	1.55	2.8238	1.6107	2.6843	1.5429
700	2.7546	1.5756	2.8382	1.6327	2.7021	1.5624
720	2.7719	1.5976	2.8529	1.6547	2.7196	1.5819
740	2.7893	1.6196	2.868	1.6767	2.7372	1.6013
760	2.8067	1.6416	2.8834	1.6988	2.7547	1.6206
780	2.824	1.6637	2.899	1.7208	2.7723	1.64
800	2.8411	1.6857	2.9148	1.7428	2.7898	1.6593
820	2.8576	1.7077	2.9307	1.7648	2.8074	1.6786
840	2.8754	1.7297	2.9469	1.7868	2.825	1.698
860	2.8933	1.7518	2.9632	1.8089	2.8425	1.7174
880	2.9115	1.7738	2.9796	1.8309	2.8601	1.7368
900	2.9298	1.7958	2.9962	1.8529	2.8776	1.7563
920	2.9483	1.8178	3.0128	1.8749	2.8952	1.7758
940	2.9669	1.8398	3.0296	1.897	2.9128	1.7953
960	2.9856	1.8619	3.0464	1.919	2.9303	1.8151
980	3.0044	1.8839	3.0633	1.9412	2.9479	1.8356
1000	3.0233	1.9059	3.0803	1.9623	2.9655	1.8571
